# Looking back on preterm birth – The successes and failures

**DOI:** 10.1111/aogs.14730

**Published:** 2024-02-14

**Authors:** Maya Reddy, Claire McGannon, Ben W. Mol

**Affiliations:** ^1^ Monash Women's Monash Health Clayton Victoria Australia; ^2^ Department of Obstetrics and Gynaecology, School of Clinical Sciences Monash University Clayton Victoria Australia; ^3^ Aberdeen Centre for Women's Health Research, Institute of Applied Health Sciences, School of Medicine, Medical Sciences and Nutrition University of Aberdeen Aberdeen UK

Preterm birth, defined as delivery before 37 weeks of gestation, remains the leading cause of childhood mortality worldwide.^1^ Annually, 13.4 million infants are born prematurely with rates of preterm birth varying between countries from 4% to 16%.^1^ Over the past two decades, advancements in neonatal care have led to a decrease in mortality associated with preterm birth. However, despite these achievements, the incidence of preterm birth remains alarmingly high, imposing a significant burden on the individual, communities, and healthcare systems.

In this issue of *Acta Obstetricia et Gynecologica Scandinavica*, Klumper et al. have explored trends in preterm birth in the Netherlands between 2011 and 2019.^2^ They demonstrated an encouraging decline in overall preterm birth from 5.45% to 5.08% in singleton pregnancies over the course of 10 years with no change to the preterm birth rates among multiple gestations. This decline was predominantly driven by iatrogenic preterm birth (from 1.77% to 1.34%) with a modest fall in the spontaneous preterm birth rates (from 3.68% to 3.62%). Importantly, these trends are marred by inequality, with no decrease in preterm birth rates in those of low socioeconomic status and women under the age of 25 years. In this editorial, we discuss the potential reasons for the decline in preterm birth observed in the Netherlands and highlight areas for improvement to further alleviate the burden of preterm birth in the years to come.

## 1 IATROGENIC PRETERM BIRTH

Over the past decade, considerable effort has been made to better predict, prevent, and manage the Four Horsemen of iatrogenic preterm birth: hypertensive disorders of pregnancy (HDP), fetal growth restriction (FGR), induction of labor for preterm prelabor rupture of membranes (PPROM), and non‐medically indicated preterm delivery. In particular, we have seen the publication of a number of landmark trials addressing optimal timing of delivery in these conditions.^3^, ^4^, ^5^ For example, in 2015, Broekhuijsen et al. demonstrated that expectant management of non‐severe HDP occurring between 34 and 37 weeks of gestation is linked to improved perinatal and neurodevelopmental outcomes with only a slight increase in adverse maternal outcomes.^3^ Similarly, in the setting of PPROM between 34 and 37 weeks of gestation, Morris et al. and van der Ham et al. have shown that immediate delivery is not associated with a decrease in neonatal sepsis rates but does lead to higher rates of adverse neonatal outcomes.^4^, ^5^ The implementation of such evidence has resulted in a shift to expectant management of those with late‐onset non‐severe disease. Considering that late‐onset preterm birth (delivery between 34 and 36 weeks of gestation) constitutes roughly 70% of all iatrogenic preterm deliveries, this shift in practice is likely to have had a significant impact on preterm birth rates in the Netherlands.^2^

In contrast to HDP and PPROM, research on the prediction, diagnosis, and prevention of FGR has been less conclusive. Furthermore, although timing of delivery for FGR before 32 weeks of gestation can be guided by sonographic biomarkers,^6^ the role of these biomarkers in guiding delivery in late‐onset disease remains uncertain. As such, it is no surprise, that Klumper et al. have shown no difference in the preterm birth rates of small‐for‐gestational‐age fetuses over the last decade.^2^ A multicenter randomized control trial addressing optimal timing of delivery in late preterm small‐for‐gestational‐age fetuses is currently underway and we remain optimistic that the results will help to address preterm birth rates within this specific population group.^7^ In the setting of HDP, emerging evidence now supports the use of prediction models for preeclampsia with prescription of low‐dose aspirin in those at high risk reducing the rates of preterm disease by 62%.^8^ Although this remains in the early stages of clinical implementation, it is likely that this intervention will also reduce the burden of preterm birth in the coming decade.

## 2 SPONTANEOUS PRETERM BIRTH

In contrast to iatrogenic preterm birth, Klumper et al. demonstrated a mere 0.06% decline in spontaneous preterm birth rates over the last decade.^2^ This could be caused by (a) the poor performance of prediction models, (b) the limited effectiveness of preventive treatments, or (c) the lack of implementation of good‐quality evidence. The two main tenets of predicting spontaneous preterm birth are maternal characteristics and transvaginal cervical length. Models that only use maternal characteristics perform poorly as most cases of preterm birth occur in those with no significant maternal history.^9^ However, there is now a large body of evidence to support the use of transvaginal cervical length to predict spontaneous preterm birth. In fact, the combination of obstetric history and mid‐trimester cervical length detects 81% of spontaneous preterm birth before 28 weeks of gestation for a 10% false‐positive rate.^10^ However, these prediction models perform poorly in late‐onset disease (spontaneous delivery between 34 and 36 weeks of gestation) and only predict 29% of such cases (false‐positive rate 10%).^10^ Late‐onset spontaneous preterm birth is far more prevalent, but the greatest impact on perinatal mortality and severe morbidity arises from delivery before 28 weeks of gestation. As such, better prediction of early‐onset disease remains an important target of screening programs and interventions.

With regards to effective interventions, multiple clinical trials have demonstrated that in those with a history of preterm birth or a short cervix, vaginal progesterone reduces the risk of preterm birth before 34 weeks of gestation by 20%.^11^ Furthermore, in those with a history of preterm birth and a short cervix (<25 mm), cerclage has also been shown to reduce the risk of delivery before 35 weeks of gestation by 30%.^12^ In those with a short cervix only, the role of cerclage remains uncertain with evidence supporting no benefit in those with a cervical length less than 25 mm.^13^ However, a subgroup analysis of five randomized control trials demonstrated that when the cervical length is less than 10 mm, cerclage may be of benefit in preventing preterm birth before 35 weeks of gestation (relative risk 0.68, 95% confidence intervals 0.47–0.98).^13^

Despite this overwhelming evidence, universal cervical length screening has not been adopted in the Netherlands and in many other countries because of concerns regarding the magnitude of the effectiveness of preventive treatments like progesterone, their cost‐effectiveness, acceptance of transvaginal ultrasound, organization of care, and the perceptions of medical practitioners.^14^ In Australia, a state‐wide implementation program that included routine mid‐trimester cervical length measurement, the use of progesterone and/or cerclage for those at risk, and the introduction of a preterm birth clinic, smoking cessation, and education about late preterm delivery, demonstrated a 7.6% reduction in overall singleton preterm birth rates.^15^ In 2012, the Israel Society of Obstetrics and Gynecology issued guidelines recommending routine mid‐trimester cervical length assessment for all women. The introduction of this guideline was associated with an overall 10% reduction in preterm birth and 24% reduction in delivery before 28 weeks of gestation.^16^

## 3 MULTIPLE PREGNANCIES

In the Netherlands, the progress made in reducing preterm birth in singleton pregnancies does not seem to extend to multiple gestations.^2^ This reflects the scarcity of evidence regarding the prediction of preterm birth in multiple pregnancies and the lack of interventions to prevent this outcome. Therefore, the opportunity to reduce preterm birth rates in this population group involves reducing the incidence of multiple pregnancies in the first place. Regulatory guidelines now recommend avoiding multiple embryo transfers during in vitro fertilization.^17^ This practice has led to a decrease in the number of multiple pregnancies over the last decade. As such, although we have not seen a reduction in preterm birth rates within this population group, the decline in absolute numbers of multiple pregnancies is likely an important contributor to the fall in preterm birth rates in the last decade.

## 4 HEALTH INEQUALITY

Unfortunately, the reduction in preterm birth rates observed by Klumper et al. does not translate to all population groups.^2^ Within the Netherlands itself, although a decline in preterm birth was observed in those of middle and high socioeconomic status, these trends were not seen in the lower socioeconomic status group. This highlights an important breakdown in the translation of evidence to clinical practice. When implementing screening programs and interventions, we must recognize the barriers to equitable outcomes within our population, be it in resource‐poor settings, culturally and linguistically diverse populations, or higher‐risk subgroups. Perhaps this highlights the importance of not only positive change, but also looking at that change through the lenses of access and engagement.

In summary, the report of Klumper et al. demonstrates that it is possible to reduce the rates of preterm birth.^2^ This requires a concerted effort in improving implementation of evidence‐informed strategies, identifying barriers to healthcare utilization, and facilitating better engagement of populations at risk of health inequality. We encourage everyone to assess where these opportunities lie in their own setting. In particular, a reduction of iatrogenic preterm birth and a critical attitude to assisted reproductive technology provide the greatest opportunity for change, with systematic screening to be a topic of future research.

## AUTHOR CONTRIBUTIONS

Maya Reddy, Ben W. Mol, and Claire McGannon were involved in the design and ideas generated in this manuscript. Maya Reddy was involved in drafting the manuscript. Ben W. Mol and Claire McGannon were involved in editing the manuscript.

## CONFLICT OF INTEREST STATEMENT

BWM is supported by an NHMRC Investigator grant (GNT1176437), reports consultancy for Merck and Organon, has received research funding from Ferring and Merck, and holds shares of ObsEva. The other authors have no conflicts of interest.
